# Book Reviews

**Published:** 1870-10

**Authors:** 


### BIBLIOGRAPHICAL NOTICES.

*The Physical Life of Woman:—Advice to the Maiden, Wife and Mother.* Sixth Edition. By Geo. H. Napheys, A. M., M. D. Author of "Compendium of Modern Therapeutics, etc., etc. Publisher, George Maclean, Philadelphia, New York, and Boston. This truly useful work, which should be in the possession of every mother, treats of the three peculiar phases of woman's life—namely, maidenhood, matrimony, and maternity. The language used is plain yet decorous, and the author deserves the gratitude of the community for thus inculcating a sound understanding of the relation of the sexes. It abounds in useful advice, and is countenanced by many distinguished physicians and divines. The fact that the fifth edition and tenth thousand was issued within three months from its first appearance, is evidence of the kind reception it has met with, and the good it will accomplish. The want of such a work as this has long been felt, and its publication demanded a degree of moral courage, which few have before this effort of Dr. Naphey's, been disposed to exhibit, through fear of the criticism which such an attempt would bring forth. But, as the author of this work truly observes, "ignorance is no more the mother of purity than she is of religion." The style in which it is gotten up is neat and attractive, and also, creditable to the publisher.

---

*The Medical Times.*—This is the title of a new semi-monthly journal of medicine and surgery, which is published by J. B. Lippincott & Co., Philadelphia and New York. Judging from the table of contents of the first two numbers which have been issued, we feel assured that this journal will rank among the best of the medical publications of our country. The yearly subscription price is four dollars, in advance.

---

"*Webster's Dictionary,*" says a Paris letter to the *Boston Post*, "is now considered throughout the continent of Europe, not only the authority *par excellence* in English lexicography, but as *the characteristic American book.* It is better known and more widely circulated than any other. I have met with it at the Imperial Library in Paris, the Library of the British Museum, the Athenæum and other London Clubs, and numerous other places. I have heard of it from Turkey, India, China, and even Japan. It is everywhere deservedly applauded for the elegance of its type, the distinctness of its impression, the beauty of the engravings, and the vast amount of information condensed within its covers. It has already taken a prominent part in moulding the English language and aiding the advance of its evergrowing empire. This result must, of course, follow from the use of a work that is found wherever our tongue is extending, as it rapidly is, through commerce and trade, among the Eastern nations. As now appears, there is no limit to its progress, and the vigilant thrift and untiring industry of the Anglo-Saxon race will insure the spread of its speech wherever their sails brighten the sluggish waters of a foreign harbor. If the language of the Bible and Shakspeare, of Burke and Macaulay, do not deteriorate in our mouth and in the utterance of those who deal with us, it will be largely owing to the onerous labors of the great Lexicographer, and the diligence of those who have so widely disseminated the evidence thereof."

